# The Psychology of Opium Eating

**Published:** 1854-04-01

**Authors:** James Bower Harrison

**Affiliations:** Corresponding Member of the Epidemielogical Society, &c. &c.


### THE PSYCHOLOGY OF OPIUM EATING.

BY JAMES BOWER HARRISON, F.R.C.S., &c.

*Corresponding Member of the Epidemielogical Society, &c. &c.*

Opium! οπος, the juice, *par excellence!* Who has not some recollection of opium? The very name brings to the mind the sick-bed of former years. The little night-draught which, with magic spell, relaxed the severity of pain, and chased away the clouds which hung over the serenity of the mind. We still remember how the kind nurse came with friendly and female care to administer the potion, and how, as night wore away, the anguish was softened—and curious faces seemed to peep round the curtains of our beds, and fancies, alien to our accustomed thoughts, mingled in our dreams until consciousness was lost, and blessed sleep for a while prevailed over the tyranny of disease. We are familiar, indeed, with these effects, but there is something very remarkable in them. That ease should be procured by the juice of a poppy! that the wonderful mind should be influenced by a cause so apparently insignificant! The great John Hunter exclaimed, Thank God for opium! and it is an undoubted blessing that the Creator should have permitted such an antidote to the sufferings of mankind.

How extraordinary is the human mind! how elevated in comprehension,—how godlike in sympathy,—and yet the human mind may be rendered joyous or fierce, wild or torpid—foolish or entranced, by such agents as alcohol or opium! Spiritual, indeed, we are, but how curiously is our spirituality mixed up with the gross and material. A miserable and despairing being, shall, under the influence of such an agent, be transferred to a paradise of joy, and yet his real condition be not a whit the less destitute. After all, there is something more in this than our philosophy can reach, but it teaches one piece of philosophy, that it is the state of the mind, rather than external circumstances, which constitutes happiness.

In this country opium is taken in the majority of instances for the purpose of obtaining sleep, or mitigating pain, or obviating the effects of exhaustion and loss of blood. But it seems also to have a singular effect on the human mind in exalting the ideas, and producing visions—an effect which has been rudely, and perhaps somewhat wrongly, compared to intoxication—of this latter property, medical writers have not entered very largely; for their experience has obviously been chiefly of its narcotic qualities, both from the mode in which they have administered it, and the intention which they have had in view. It seems that if opium is taken in comparatively small and frequently repeated doses, it produces excitement and pleasurable feelings before it occasions stupor. The capability of receiving excitement from it is probably increased by habit, somewhat in the same manner that alcoholic liquors give most pleasure to those who are in some degree habituated to them. Certain constitutions are, also, no doubt more favourable to the production of these effects than others. It is only by such considerations that the surprising effects related of opium eating in the East, can be reconciled to the experience of the profession at large in this country. But if the effects of opium are thus pleasurable in the first instance—the necessity of continuing the stimulus—the slavery of habit (the most abject of all slaveries) and the degradation and wretchedness which eventually ensue, are a terrible punishment. How dreadful the tyranny of a habit which insensibly coils itself, like a deadly snake, round the victim which it fascinates, until escape is impossible. Education, talents, refinement of mind, all are in vain—the embrace of the destroyer is in too many folds to be untwisted;—at length the fascination is gone, and the glaring eyes of a fiend are upon him for ever. Even sleep—that balm of hurt minds—that nurse of nature—that chief nourisher in life's feast—even sleep is gone, and all the pure affections are poisoned, or turn to bitterness;—the simplicity of children,—the love of woman,—the peacefulness of religion—they are no more.

We shall consider these effects chiefly as they are evidenced in two memorable instances, which are, indeed, the type of others and of all. Every one knows that in the East, the exhilarating properties of opium have been greatly abused. Mr. Madden, in his "Travels in Turkey," &c., gives a brief description of the opium eaters in Constantinople. The coffee-houses in which they assemble, are situated in a large square, and on the benches outside the door they sit and indulge in the reveries to which the drug gives rise. He states that their gestures were wild, their features flushed, and their talk incoherent. Some, however, addressed eloquent discourses to the bystanders, and others appeared to be enjoying the most beatific ideas. Mr. Madden was himself desirous of experiencing the effects. He first took one grain of opium, but an hour and a half elapsed without any perceptible effect. The keeper of the coffee-house wished to give him two grains more, but he only consented to half this quantity. However, he subsequently took an additional quantity of two grains, and then he became sensibly excited. Everything now appeared enlarged in volume—there was a sort of curious expansion of mind and matter. But Mr. Madden discovered that the pleasure was chiefly derived from external objects, and that when he closed his eyes the same feelings were no longer excited. He now determined to make his way home as fast as possible, but as he went he feared to commit some extravagance. He was hardly sensible that his feet touched the ground, but seemed to slide along as if propelled by some invisible agency, which rendered his body lighter than the air. The moment he got home he went to bed, but the same delightful visions filled his mind all the night. The next day, however, he rose pale and dispirited, with headache and feebleness, so that he was all that day confined to the sofa. Mr. Madden speaks of the practice as extremely injurious to the opium eaters themselves—they lose their appetites—become feeble and tremulous—their necks wry, and their fingers contracted—they are perfectly miserable until the hour arrives for the gratification of their indulgence. Dr. Oppenheim, a German writer, makes a similar statement—"The habitual opium eater," says he, "is instantly recognised by his appearance—a total attenuation of body, a withered yellow countenance, a lame gait, a bending of the spine, frequently to such a degree as to assume a circular form, and glassy deep sunken eyes, betray him at first glance."* Dr. Oppenheim mentions that the habit is almost impossible to break, but those who make the attempt, ingeniously mix their pills with wax, and daily diminish the quantity of opium until nothing but the wax remains.—But I shall now pass on to give some notice of the life of a great poet, Samuel Taylor Coleridge, a man of uncommon talent and extensive learning. Perhaps it may be remembered that he was the author of that wild but beautiful poem, the "Ancient Mariner," which begins in this curious strain:—

"It was an Ancient Mariner,
And he stoppeth one of three;
By thy long gray beard, and glittering eye,
Now, wherefore, stopp'st thou me."

I shall not pretend to give any regular account of the life of Coleridge, but content myself with such few particulars as may give interest to what follows:—Coleridge was born in Devonshire, and was the youngest son of the Rev. John Coleridge, who was the Vicar of the parish of St. Mary Ottery, his native place.

* See Pereira, "Materia Medica," vol. ii. p. 1746.

His education was first conducted at Christ's Hospital, and subsequently at Cambridge, under the Rev. James Bowyer. There is something singular in the fact, that Mr. Coleridge, like Mr. De Quincey, ran away from his scholastic pursuits. During the time that Coleridge was at Cambridge, he fell in love with a young woman, who rejected his addresses. This produced so much effect upon his mind that, in a fit of despondency, he ran away to London. Here he enlisted as a common soldier, in a regiment of horse, assuming the somewhat awkward name of Silas Tomken Cumberbatch. Mr. Coleridge was far from acquitting himself well in this new capacity. He was unable to rub down his horse with credit, and is said to have been assisted by a companion, in return for which service, he wrote love stanzas, that his friend might appear well in the eyes of his sweetheart. He did not succeed much better as a rider than as a groom, and sometimes, to the amusement of his associates, in mounting on one side of his horse he fell over on the other. The manner in which he got extricated from his military service is on a par with the rest of his adventures. One day he happened to hear some of the officers quoting, or rather mis-quoting, a passage of Euripides, and touching his cap, he ventured, in a very respectful manner, to set them right. This, of course, led to inquiry as to his former life, and in the end he was taken to the medical department at the hospital, from which his friends ultimately removed him. Mr. Coleridge, in his literary biography, gives us an amusing picture of his early efforts to establish himself in life. He was persuaded, in the first instance, to commence a periodical under the title of the "Watchman," and set out to Birmingham to solicit subscriptions. He had first an interview with a tallow-chandler, to whom he expatiated on his project. But the tallow-chandler was a man on whom all the rhetoric of Coleridge was lost. "And what, sir," he said, after a pause, "might the cost be?" "Only fourpence," replied Coleridge; "and O!" he adds, "how I felt the anti-climax, the abysmal bathos of that fourpence. Only fourpence each number, to be published on every eighth day." "That comes," said the chandler, "to a deal of money at the end of the year, and how much was there to be for the money?" "Thirty-two pages, sir, large octavo, closely printed—thirty-and-two pages." "Bless me! why, except what I does in a family way on the Sabbath, that's more than I ever reads, sir, all the year round. I am as great a one as any man in Birmingham, sir, for liberty and truth and all them sort of things; but as to this (no offence, I hope, sir,) I must beg to be excused." Coleridge soon found that to be an author by profession is to live a most arduous as well as unprofitable life, and he writes feelingly upon this point in the way of advice to others. He had soon an amusing proof of the unsaleableness of his own writing, for rising one morning earlier than usual, he found the servant girl lighting the fire with an extravagant quantity of paper. On offering a gentle remonstrance, poor Mary replied, "La! sir! why, it's only the *Watchman*." I need not here enter into a systematic account of the various publications which established the fame of Coleridge; the success of his literary and poetical career is sufficiently known to the world. But talents and learning do not ensure happiness nor prosperity. The excitement of genius is not always compatible with the tranquillity of domestic life, nor always consistent with the steady progress of pecuniary advancement. The subtleties of metaphysics, and the grandeur of poetical conceptions, did not avail Coleridge in the acquisition of fortune. He began to experience the pressure of poverty, but he also experienced a greater misfortune in seeking to restore his bodily and mental energies by recourse to opium. To how great an extent he carried this habit will shortly appear from some letters which are published by his friend, Mr. Cottle, in his "Early Recollections of Coleridge." Mr. Cottle apologises for offering these letters to the public, on the ground of their extreme value; and indeed it was the expressed wish of Coleridge that his example should, as far as possible, be made a warning to others. Mr. Cottle states, that as soon as he suspected the real nature of Mr. Coleridge's misfortunes, and their connexion with his practice of opium eating, he wrote him a long and earnest letter, begging him to renounce the dreadful habit; and so greatly was Mr. Cottle struck with the importance of the revelations to which his letter led, that he says, speaking of his account of Mr. Coleridge's infirmity—"It is consolatory to believe, that had I written nothing else, this humble but unflinching narrative would be an evidence that I had not lived in vain."* The following is the reply which Mr. Coleridge addressed to Mr. Cottle:—

"*April 26th*, 1814.—You have poured oil in the raw and festering wound of an old friend's conscience, Cottle! but it is oil of vitriol! I but barely glanced at the middle of the first page of your letter, and have seen no more of it—not from resentment (God forbid!) but from the state of my bodily and mental sufferings, that scarcely permitted human fortitude to let in a new visitor of affliction. The object of my present reply is, to state the case just as it is—first, that for ten years the anguish of my spirit has been indescribable, the sense of my danger staring, but the consciousness of my guilt worse, far worse, than all! I have prayed with drops of agony on my brow; trembling, not only before the justice of my Maker, but even before the mercy of my Redeemer. 'I gave thee so many talents, what hast thou done with them?' Secondly, overwhelmed as I am with a sense of my direful infirmity, I have never attempted to disguise or conceal the cause. On the contrary, not only to friends have I stated the whole case with tears, and the very bitterness of shame; but in two instances I have warned young men, mere acquaintances, who had spoken of having taken laudanum, of the direful consequences, by an awful exposition of its tremendous effects on myself. Thirdly, though before God I cannot lift up my eye-lids, and only do not despair of his mercy, because to despair would be adding crime to crime, yet to my fellow men I may say, that I was seduced to the accursed habit ignorantly. I had been almost bed-ridden for many months with swelling in my knees. In a medical journal, I unhappily met with an account of a cure performed in a similar case (or what appeared to me so) by rubbing in of laudanum, at the same time taking a given dose internally. It acted like a charm, like a miracle! I recovered the use of my limbs, of my appetite, of my spirits, and this continued for near a fortnight. At length the unusual stimulus subsided, the complaint returned,—the supposed remedy was recurred to—but I cannot go through the dreary history. Suffice it to say, that effects were produced which acted on me by terror and cowardice of pain, and sudden death, not (so help me God!) by any temptation of pleasure, or desire of exciting pleasurable sensations. On the very contrary, Mrs. Morgan and her sister will bear witness so far as to say, that the longer I abstained, the higher my spirits were—the keener my enjoyments—till the moment, the direful moment arrived, when my pulse began to palpitate, and such a dreadful falling abroad, as it was, of my whole frame, such intolerable restlessness and incipient bewilderment, that in the last of my several attempts to abandon the dire poison, I exclaimed in agony, which I now repeat in seriousness and solemnity—'I am too poor to hazard this.' Had I but a few hundred pounds; but £200, half to send to Mrs. Coleridge, and half to place myself in a private *mad-house*, where I could procure nothing but what a physician thought proper, and where a medical attendant could be constantly with me for two or three months (in less than that time life or death would be determined), then there might be hope—now there is none!! O God! how willingly would I place myself under Dr. Fox, in his establishment; for my case is a species of madness, only that it is a derangement, an *utter impotence of the volition, and not of the intellectual faculties*. You bid me rouse myself; go bid a man paralytic in both arms to rub them briskly together, and that will cure him. 'Alas!'"

* "Early Recollections," page 138.

he would reply, 'that I cannot move my arms is my complaint and my misery.' May God bless you, and your affectionate but most afflicted—S. T. COLERIDGE."

In Mr. Coleridge's account of his melancholy state, we have an admirable description of the peculiar condition into which certain minds may be brought by the influence of habit. He has happily expressed a psychological truth—that there is a form of insanity, or infirmity of mind, which consists in an utter *impotence of volition:* the patient himself is anxious to escape the dominion of some particular propensity, and is alive to the imbecility under which he labours. Thus, many have felt compelled to shelter themselves under the protection of stronger or better regulated minds, and even found satisfaction in yielding their liberty for the safety they acquired in return. Perhaps no subject is of more importance than that of the dominion of habit. It is strange to think that the strongest habits have been built up by separate and isolated instances. We suppose that we can do wrong as long as we choose, and withdraw unhurt. We contemplate the monster afar off, whilst the infernal web is being spun around us, and when we seek to retire we are engaged in its interminable toils. Each act by which the habit was acquired was of our own free will, but being acquired, our will seems suspended. The acts are then half involuntary, and the mind is only partially cognizant of them, or impotent to oppose them. Happily in some few cases strong and well directed efforts break the chains which bind the victim, but more often the mind sinks in weak and ineffectual struggles—contemplating its own misery whilst passing into the jaws of destruction. Yet there is this consolation in the law of habit, that it may lead to good as well as bad results.

"That monster custom, who all sense doth eat,
Of habit's devil, is angel yet in this :
That to the use of actions fair and good
He likewise gives a frock or livery
That aptly is put on."

It would be a curious subject to speculate as to the number of repetitions which are necessary to constitute a habit, but however interesting such speculation might be, we must here return to the unfortunate Coleridge. In another letter, he says,—"Dear Cottle,—I have resolved to place myself in any situation, in which I can remain for a month or two, as a child, wholly in the power of others. But alas! I have no money! Will you invite Mr. Wood (a most dear and affectionate friend to worthless me); and Mr. Le Breton, my old school-fellow, and likewise a most affectionate friend; and Mr. Wade, who will return in a few days; desire them to call on you any evening after seven o'clock that they can make convenient, and consult with them whether anything of this kind can be done. Do you know Dr. Fox? Affectionately, S. T. C.

"I have to prepare my lecture, oh! with how blank a spirit!"*

It is indeed lamentable to see the fine talents of Coleridge thus reduced, and his very capability of writing rendered abortive by internal misery. "I cannot" (says he, in one place) "as is feigned of the nightingale, sing with my breast against a thorn." We see him with health destroyed, money wasted, and domestic happiness sacrificed, oppressed with debt, and with independence gone. He who carried away prizes at the University, and was the admiration of all who could estimate genius. Who shall say he is safe, if genius can thus succumb? His "tottering step and glassy eye" told of the miserable servitude into which habit had drawn him. Sir Humphrey Davy had well described the instability of his mental constitution, when he compared "the brilliant images of greatness which floated on his mind" to the images of morning clouds mirrored on the waters, "which are agitated by

* "Early Recollections," page 162.

every breeze, and modified by every sunbeam." It may be supposed that strenuous efforts were made by Mr. Coleridge's friends to reclaim him. Medical assistance was procured, and by the kind intervention of Mr. Josiah Wade, of Bristol, a respectable person was procured to live with him, and exercise a constant surveillance over him, both by night and by day. But even this plan failed, for, as Mr. Coleridge confessed afterwards, he managed still to obtain the laudanum by secret and dexterous means. On one occasion as he was passing along a quay with his attendant, he pointed to a ship, and requested the man to see whether it was an American vessel. The man assured him that it was not, but being requested to step over and ascertain, he left Mr. Coleridge for a short time, during which Mr. Coleridge ran to a druggist's and obtained a supply of laudanum in a bottle, which he always carried in his pocket. Amongst the kind friends who generously aided Mr. Coleridge with pecuniary assistance, was Mr. De Quincey, the well-known author of the "Confessions of an Opium Eater." Mr. De Quincey early discovered the talents of Mr. Coleridge, and learning from Mr. Cottle that he was in embarrassed circumstances, at once offered him £500. Mr. Cottle thought the sum too large to be presented in the first instance, and it was finally arranged that £300 should be given. Mr. De Quincey, with the delicacy characteristic of his gifted mind, desired that his own name should not transpire, and that the present should be made as coming from an unknown admirer of the genius of Coleridge.—The quantity of laudanum which Mr. Coleridge took was amazingly large, and consequently the expense considerable. For years, the purchase of opium had exceeded £2. 10s. per week. He was in the habit of taking from two quarts of laudanum a week to a pint a day; and on one occasion he had been known to take a quart of laudanum in twenty-four hours.* These statements would almost appear incredible, even upon the respectable authority of Mr. Cottle, were it not for some similar accounts given by the distinguished toxicologist, Dr. Christison, and the late eminent Dr. Pereira.—I must be pardoned one more quotation, for the following letter is so valuable that I cannot bring myself to omit it. It is addressed to Mr. Wade, and is dated Bristol, June 26th, 1814,—"Dear sir, for I am unworthy to call any good man friend, much less you, whose hospitality and love I have abused; accept, however, my entreaties for your forgiveness and your prayers. Conceive a poor miserable wretch, who for many years has been attempting to beat off pain by a constant recurrence to the vice that reproduces it. Conceive a spirit in hell, employed in tracing out for others the road to that heaven from which his crimes exclude him! In short, conceive what is most wretched, helpless, and hopeless, and you will form as tolerable a notion of my state as it is possible for a good man to have. I used to think the text in St. James, that 'he who offended in one point offends in all,' very harsh; but now I feel the *awful, the tremendous truth of it.* In the one *crime of opium,* what crime have I not made myself guilty of? Ingratitude to my Maker! and to my benefactors—injustice! and unnatural cruelty to my poor children! Self-contempt for my repeated promise-breach, nay, too often actual falsehood! After my death, I earnestly entreat that a full and unqualified narration of my wretchedness, and of its guilty cause, may be made public, that, at least, some little good may be effected by the direful example! May God Almighty bless you, and have mercy on your still affectionate and, in his heart, grateful,—S. T. COLERIDGE."†

This letter is worthy of being preserved, if for no other, for this reason, that it bears evidence of the sacred truth—that if we would be virtuous and happy, we must make no exception for a favourite vice—for a *venial* fault—

* "Early Recollections," p. 169.

† It is pleasing to be able to state that Coleridge eventually overcame the habit of opium taking.

one break in the harmony of virtue, and the whole is unhinged—one link unfastened, and the whole chain falls into pieces. Let no one think that he will be good, *with one exception*. If we offend in one point, we shall soon offend in all, for the fine sense of right is gone, and the integrity of virtue can bear no division. Coleridge died on the 25th of July, 1834, having written for himself the following epitaph.

> "Stop, Christian passer-by! Stop, child of God!
> And read with gentle breast.—Beneath this sod
> A poet lies, or that which once seemed he;
> Oh, lift a thought in prayer for S. T. C.!
> That he who many a year with toil of breath
> Found death in life, may here find life in death!
> Mercy for praise—to be forgiven for fame,
> He asked and hoped through Christ—Do thou the same."

It is somewhat remarkable, that one who so destroyed the serenity of his own natural sleep by narcotic drugs, should be the author of these beautiful lines,—

> "O sleep! it is a gentle thing,
> Beloved from pole to pole.
> To Mary, Queen, the praise be given:
> She sent the gentle sleep from heaven
> That slid into my soul."

The effect of habitual opium taking, on health and longevity, has been a subject of legal consideration. A remarkable trial took place in relation to some assurances effected by the late Earl of Mar in the Edinburgh Life Assurance Company. The company discovered, on the death of the earl, that he had been in the habit of taking opium to a large amount, and, on that ground, refused to pay the insurance. The case was decided against the company on the presumption that they had not been sufficiently careful in their preliminary inquiries as to his habits. Dr. Christison, who was concerned as a medical witness in this case, was led, in the course of his investigation, to some interesting data, both in respect to the frequency of this habit, and its effect on the duration of life. It must be confessed that, from these inquiries, opium does not seem so rapidly destructive as might be supposed; but there is no revelation made as to the misery in which life was passed; and, in all probability, a vast number of fatal cases have been in more than one sense *buried* in oblivion. However, Dr. Christison's cases are replete with interest, and will be read by those who are concerned in similar inquiries with the greatest advantage. He gives a short statement of the ages of the opium eaters, and the quantities of opium taken. It would appear that many reached advanced periods of life, such as fifty or sixty, after fifteen, twenty, or thirty years of this lamentable practice. One old woman, who died at Leith at the age of eighty, had taken half an ounce of laudanum daily for nearly forty years, and enjoyed tolerably good health all the time. Another, who died at seventy-six, had taken about the same quantity, and for the same time. Very many such statements are made, but I conceive they are exceptions from a general rule, and that the health was by no means so good as was represented; for in some instances these persons are stated to have given up the opium for intervals, which they would scarcely have done if it had contributed to pleasure without impairing the health. Dr. Christison must be himself aware that a long list of drunkards might be made who had escaped the evils consequent on their habits, and who have died at an advanced age. Such statements, however, are interesting chiefly as matters of curiosity; and the example, as the poet says, "more honoured in the breach than the observance."

The next page in the history of opium eating is revealed in the "Confessions of an Opium Eater." This extraordinary book is written in so pleasing a style, and so nicely blended with narrative, that it is impossible not to be interested with it. The writer, De Quincey, is evidently a man of highly cultivated mind, and of vivid imagination, and has invested the subject of opium with all the charms of elegant composition and powerful delineation. But we cannot avoid feeling persuaded that, in the retrospect of his life and of his feelings, he has given too poetical a colouring to the picture, or at least kept subdued in the background those more repulsive features which startle us in the confessions of Coleridge. Endowed with a fertile mind, and richly stored with the treasures of learning, he had a more than usual proclivity to ideal pains and pleasures; but he has passed over the *common-places* of misery, the degradation of mind which habit imposes, the horror and revulsion of feeling which arise from a perpetual interference with the simplicity of the natural affections. He has touched with a graphic pen the dreams and visions which he experienced; but he has not dwelt on the days of debasing and tremulous prostration which wait on the excitement. He has given, in effect, an air of romance to all, and, with unusual skill, blended his narrative with scenes of exquisite pathos. But for this very reason we are constrained to remember that this story has more of the gloss of fiction than the terror of reality.

When about seven years of age, the opium eater lost his father, and was committed to the care of guardians. They sent him to various schools, and it appears that he obtained a good education, and made considerable progress, especially in the Greek language. As he grew older he was desirous of being sent to college, but in this wish he was not permitted to indulge. The disappointment acting powerfully on his mind, he determined to run away from school; and, about the commencement of his seventeenth birthday, proceeded to put his resolve into execution. Not having money sufficient to carry out his views, he wrote to a lady of rank who had known him from childhood, requesting the loan of five guineas. In answer to this letter, she sent him ten, which immediately decided him to enter upon his adventure. It was not, however, without a sorrowful feeling that he quitted the scene of his youth. "On the evening before I left," says he, "I grieved when the ancient and lofty school-room resounded with the evening service, performed for the last time in my hearing; and at night, when the muster-roll of names—and mine as usual was called first—I stepped forward, and passing the head master, who was standing by, I bowed to him, and looked earnestly in his face, thinking to myself—'He is old and infirm, and, in this world, I shall not see him again." I was right; I never *did* see him again, nor ever shall. He looked at me complacently, smiled goodnaturedly, returned my salutation (or rather valediction), and we parted, though he knew it not, for ever." The next day he rose at half-past three: it was a beautiful July morning, and there was something which affected him in the quietude of that early hour, with the broad but softened light which shed itself on the adjacent towers. A picture hung over his mantlepiece of a beautiful countenance, which he had often gazed at with a sort of devotion. As he was looking at this picture for the last time, the clock struck four; he went to the picture, kissed it, and gently walked out. He was not destined, however, to make his exit so quietly as he had expected. It was necessary to move a large trunk, which was too heavy to be carried by his own unaided exertion. A servant man had kindly offered to assist him,—a man—

> "Of Atlantean shoulders, fit to bear
> The weight of mightiest monarchies,"

but the man had the misfortune to slip, and the trunk fell and rolled with great impetus against the door of the pedagogue. For a time they thought that all was lost, but, curiously enough, the doctor, who was generally a light sleeper, never awoke. He was now launched out on the world. It will easily be supposed that his resources would soon become inadequate to his wants. For some time he wandered about in the mountainous parts of Wales; and at one time supported himself by writing letters for cottagers who happened to have friends at a distance. Once he was entertained some days by a family of young people, for whom he acted as correspondent, and gave great satisfaction by the delicate manner in which he indited love letters for a kind and amiable girl. The parents of these people, however, returning, put an end to his continuance with them. From Wales he contrived to get to London, though he omits to state in what manner; and here his sufferings began in earnest. For upwards of sixteen weeks he was a prey to the most bitter hunger. He slept for a long time in the open air, and subsisted on a precarious charity. At length an individual permitted him to take shelter in an unoccupied house, and there, with a friendless and deserted child, on whom he took compassion, he passed weary days and nights. It seems singular that in this destitute state he did not again have recourse to the protection of his guardians; and he does not give sufficient reasons for his not doing so, as he nowhere states that he was treated by them with any excess of severity. It is probable, however, that a want of sufficient resolution, and a certain dread of again losing his liberty, prevailed over other feelings. It is about this period of his life that he introduces us to a little episode in his history, which is told with such touching simplicity, that it is with reluctance I am led to abridge it. In wandering in the streets of London by night, he had formed a sort of companionship with an unfortunate girl. They sought each other regularly at an appointed place; and her companionship was the solace of his miserable life. The youth of the girl, and the interest she displayed in his misfortunes, gave rise to an attachment of the strongest nature. He never knew more than her Christian name, and, as he always depended upon finding her, he did not think it necessary to learn more.

It happened one day that the opium eater met, casually, with a friend in Albemarle Street, and being recognised, related his history. His friend supplied him with a small sum of money, with which he resolved to visit Eton to see the son of a nobleman, with whom he was acquainted, and through whose means he hoped to effect some monied arrangement on the strength of his expectations. He took leave of Anne (for that was the name of the young woman) as usual, never doubting but that he should find her on his return. When he came back he hastened to the accustomed place, anxious to make known the success of his enterprise, and share with her his amended fortune. In vain he looked amongst the busy throng by the lamplight of Oxford Street. They had parted for ever. Perhaps she was at the very time in search of him also; perhaps a street only divided them. "O, Oxford Street," exclaims he, "stony-hearted step-mother! thou that listenest to the sighs of orphans, and drinkest the tears of children! successors of myself and Anne have doubtless since trodden in our footsteps; and thou, Oxford Street, hast since echoed to the groans of innumerable hearts." After this relation, we are introduced by the opium eater to the commencement of his terrible habit. He caught a violent pain in the head and face from an imprudent application of cold water, and was recommended by a college acquaintance to take opium. This he immediately purchased, and was delighted with the ease he obtained, and the agreeable feelings it produced. He was charmed at the idea that pain could be so cheaply assuaged, and his mind pleasantly excited. He soon became habituated to the stimulus, and thought himself happy in its discovery. Life seemed to have gained new charms, and to present itself in new aspects. Under the influence of opium he saw with a different sight, and heard with different ears. As he went out and mixed with the busy throng of London, all seemed to wear a fresh and beautiful appearance. At the opera the scene became actually a paradise, the strains of music were heavenly, and the spectacle like a fairy enchantment; even common things lost their grossness; in fact all was seen and felt through a new medium. He wandered in the streets of London whilst under this influence, and took pleasure in everything which surrounded him; for motion itself was pleasure. I may here remark that the opium eater finds fault with the statements which are generally made with respect to opium. He denies that it occasions intoxication, and he is doubtless correct in objecting to this term being applied without due qualification. The pleasure of wine is one that rises to a certain pitch, and then declines or degenerates into stupidity; while that of opium, he asserts, remains stationary for eight or ten hours. Again the influence of wine is of a nature to disorder the mind, whilst opium tends to exalt the ideas, and yet contribute to harmony and order in the arrangement. Nor do we find that *maudlin* character in the excitement of the moral feelings consequent on opium, which so often renders the inebriated an object of ridicule. He further denies that opium produces that subsequent depression which is commonly supposed to follow excitement. He remarks that, in his own case, he always felt unusually hilarious on the day following its enjoyment. In these statements, however, it must be allowed that there is not perhaps that absolute contradiction of medical authority which he supposes. The term *intoxication* may or may not be extended to embrace the ideas of the opium eater, according to the latitude of the definition; and the individual experience of a mind, prone to excitement, cannot be regarded as a certain test of the manner in which others may be affected. Yet it is probable that the ordinary representations of medical writers are somewhat incorrect, and the experience of the profession as to the exciting influence of opium not equally extensive with that of its narcotic and poisonous effects.

Some melancholy event having occurred in the year 1813, tended to confirm the opium eater in his practice of opium-eating, and he soon found the habit so strong that it was almost impossible to avoid the indulgence. Certain uneasy feelings in his stomach also rendered it difficult to tolerate any abstinence from it; and now he began to experience something of the tyranny of the drug. The boundary between his waking and sleeping thoughts seemed broken. The minutest events of his past life came across his mind—his dreams were vivid and terrible, and the ideas which had passed through his mind presented themselves again in fantastic shapes and grotesque figures. But the horrible predominated, and he began to fear sleep. Perhaps, as he somewhere observes, nothing which is once written on the brain is ever actually destroyed. May it not reappear hereafter, as the stars come again into sight when the daylight is gone from the heavens? Some idea of the nature of his dreams may be gathered from the curious notices which he has preserved. From the character of his previous studies, mythological or oriental scenes often tyrannized over his imagination. "From kindred feelings," says he, "I soon brought Egypt and all her gods under the same law—I was stared at, hooted at, grinned at, chattered at, by monkeys, by paroquets, by cockatoos —I ran into pagodas, and was fixed for centuries at the summit or in secret rooms—I was the idol—I was the priest; I was worshipped, I was sacrificed— I fled from the wrath of Brama through all the forests of Asia—Vishnu hated me; Seeva laid wait for me; I came suddenly upon Isis and Osiris—I had done a deed, they said, which the ibis and the crocodile trembled at—I was bound for a thousand years in stone coffins, with mummies and sphinxes, in narrow chambers, at the heart of eternal pyramids. I was kissed with cancerous kisses by crocodiles, and laid confounded with all unutterable slimy things, amongst reeds and Nilotic mud. * * * Over every form, and threat, and punishment, and dim, sightless incarceration, brooded a sense of eternity and infinity that drove me into an oppression as of madness. Into these dreams only it was, with one or two slight exceptions, that any circumstance of physical horror entered. All before had been moral and spiritual terrors. But here the main agents were ugly birds, or snakes, or crocodiles, especially the last. The cursed crocodile became to me the object of more horror than almost all the rest. I was compelled to live with him; and (as was always the case almost in my dreams) for centuries. I escaped sometimes and found myself in Chinese houses, with cane tables, &c. All the feet of the tables, sofas, &c., soon became instinct with life; the abominable head of the crocodile, and his leering eyes, looked out at me, multiplied into a thousand repetitions, and I stood loathing and fascinated. And so often did this hideous reptile haunt my dreams, that many times the very same dream was broken up in the very same way. I heard gentle voices speaking to me (I hear everything when I am sleeping); and instantly I awoke: it was broad noon; and my children were standing hand in hand, at my bedside, come to show me their coloured shoes or new frocks, or let me see them dressed for going out. I protest, that so awful was the transition from the damned crocodile, and the other unutterable monsters and abortions of my dreams, to the sight of innocent *human* natures, and of infancy, that in the mighty and sudden revulsion of mind, I wept, and could not forbear it, as I kissed their faces." Old scenes would often come across his mind, like the sailing clouds across the sky; sometimes he fancied he was walking in pleasant pastures, and lanes of quiet beauty; and then the picture would change to grander and more imposing objects. Once he thought it was an Easter Sunday, and that he was by his cottage door, and the hedges were rich with roses, and in the green churchyard cattle were quietly grazing, and as he turned to open his garden gate, the scene changed to one of oriental character:—"At a vast distance were visible," says he, "as a stain upon the horizon, the domes and cupolas of a great city; an image, or faint abstraction, caught, perhaps in childhood, from some picture of Jerusalem, and not a bow-shot from me, upon a stone, shaded by Judean palms, there sat a woman, and I looked, and it was—Anne! She fixed her eyes upon me earnestly, and I said to her, at length—'So I have found you at last.' I waited, but she answered not a word; her face was the same as when I saw it last, and yet again how different! Seventeen years ago, when the lamp-light fell upon her face, and for the last time I kissed her lips, her eyes were streaming with tears; the tears were now wiped away; she seemed more beautiful than she was at that time, but in all other points the same, and not older. Her looks were tranquil, but with unusual solemnity of expression; and now I gazed upon her with some awe; but suddenly her countenance grew dim, and turning to the mountains I perceived vapours rolling between us: in a moment all had vanished; thick darkness came on, and in the twinkling of an eye, I was far away from mountains, and by lamp-light in Oxford-street, walking again with Anne, just as we walked seventeen years before, when we were both children." The transformations and variations of these ideal pictures remind us of those dissolving scenes which show us castles turning into landscapes, or trees becoming ships on the expanded ocean. But here and there, amidst the inconsistencies of imaginary things, arises some incident of life, which, seen for a while in its natural beauty, with all the affecting reminiscences of the past, grows suddenly distorted in proportions, and loses itself in frightful forms of squalid poverty and garish misery. Another dream is still more exciting, and will be excused as a further and a graphic delineation of these opiate reveries. "The dream commenced with music, which now I often heard in dreams—a music of preparation and of awakening suspense; a music like the opening of the Coronation Anthem, and which like that, gave the feeling of a vast march—of infinite cavalcades filing off, and the tread of innumerable armies. The morning was come of a mighty day, a day of crisis and of final hope for human nature, then suffering some mysterious eclipse, and labouring in some dread extremity. Somewhere, I know not where—somehow, I know not how—by some beings, I know not whom—a battle, a strife, an agony, was conducting, was evolving like a great drama, or piece of music, with which my sympathy was the more insupportable from my confusion as to its place, its cause, its value, and its possible issue. I, as is usual in dreams (where, of necessity, we make ourselves central to every movement), had the power, and yet had not the power, to decide it. I had the power if I could raise myself, to will it; and yet again had not the power, for the weight of twenty Atlantics was upon me, or the oppression of inexpiable guilt, 'deeper than ever plummet sounded.' I lay inactive. Then, like a chorus, the passion deepened; some greater interest was at stake, some mightier cause than ever yet the sword had pleaded, or trumpet had proclaimed. Then came sudden alarms; hurryings to and fro; trepidations of innumerable fugitives, I knew not whether from the good cause or the bad; darkness and lights, tempests and human faces, and at last, with the sense that all was lost, female forms, and the features that were worth all the world to me, and but a moment allowed,—and clasped hands, and heart-breaking partings, and then,—everlasting farewells! and with a sigh, such as the caves of hell sighed when the incestuous mother uttered the abhorred name of death, the sound was reverberated—everlasting farewells! and again, and yet again reverberated—everlasting farewells!

"And I awoke in struggles, and cried aloud, 'I will sleep no more!'" How truly might he have said,

"Macbeth hath murdered sleep!
Macbeth shall *sleep no more!*"

The efforts of the opium-eater to renounce the practice are extremely interesting, and it is to be regretted that he has not written more on *this*, the most important part of the subject. Every one knows something of the pertinacity with which habits remain. The diary which is here given is a singular document, and tells the number of drops of laudanum taken daily during four weeks. It is as follows:—

| FIRST WEEK. | |
|---|---|
| Monday, June 24 ...... | 130 |
| ,, 25 ...... | 140 |
| ,, 26 ...... | 130 |
| ,, 27 ...... | 80 |
| ,, 28 ...... | 80 |
| ,, 29 ...... | 80 |
| ,, 30 ...... | 80 |
| **SECOND WEEK.** | |
| Monday, July 1 ...... | 80 |
| ,, 2 ...... | 80 |
| ,, 3 ...... | 90 |
| ,, 4 ...... | 100 |
| ,, 5 ...... | 80 |
| ,, 6 ...... | 80 |
| ,, 7 ...... | 70 |

| THIRD WEEK. | |
|---|---|
| Monday, July 8 ...... | 300 |
| ,, 9 ...... | 50 |
| ,, 10 | Hiatus in MS. |
| ,, 11 | |
| ,, 12 | |
| ,, 13 | |
| ,, 14 ...... | 76 |
| **FOURTH WEEK.** | |
| Monday, July 15 ...... | 76 |
| ,, 16 ...... | 73½ |
| ,, 17 ...... | 73½ |
| ,, 18 ...... | 70 |
| ,, 19 ...... | 240 |
| ,, 20 ...... | 80 |
| ,, 21 ...... | 350 |

In this detail the relapses will be viewed as the most curious part. But how natural are such relapses to the frailty of human kind! The gnawing sensations which attended a diminution of the opium were almost intolerable, and it was from these feelings, rather than from mental depression, that the difficulty of abandoning it arose.* If we consider the monotony of some

* The writer of an article on Mental Dietetics in the fourth volume of this Journal, says:—"We may truly say that Mr. De Quincey is one of the most remarkable men we had ever the pleasure of meeting: his conversation is always characterised by the clearest reasoning and the happiest choice of language; he is a profound Greek lives—the anxiety and incessant labour of others—the heart-rending trials which occur in most, and the utter desolateness and despair of a few, it would indeed be a great boon to humanity, if, without crime, without moral and physical degradation, without sinning against the great Creator, it were possible by some means to cheat the mind of its own wretchedness—to forget, even for a time, the evils of the day—"the oppressor's wrong, the proud man's contumely, the insolence of office, and the spurns that patient merit of the unworthy takes." It would be a great boon if it were possible to exalt at will the energies of the mind—to clothe with the treasures of intellectual grandeur the ordinary events of the passing hour, and give increased refinement to every emotion of the heart. But virtue is sacrifice; we cannot thus evade the trials of life, and anticipate a felicity for which our nature is unprepared. We may indeed have a glorious excitement; but soon a feverish perturbation occupies our waking hours, and fearful dreams make horrible our pillows. Then let

"The frame of things disjoint,
Both the worlds suffer,
Ere we will eat our meal in fear, and sleep
In the affliction of those terrible dreams
That shake us nightly."—*Macbeth.*

*Higher Broughton, near Manchester.*

---

